# Effect of varying UAV height on the precise estimation of potato crop growth

**DOI:** 10.3389/fpls.2023.1233349

**Published:** 2023-08-17

**Authors:** Stephen Njehia Njane, Shogo Tsuda, Bart M. van Marrewijk, Gerrit Polder, Kenji Katayama, Hiroyuki Tsuji

**Affiliations:** ^1^ Hokkaido Agricultural Research Center, National Agriculture and Food Research Organization, Memurocho, Kasaigun, Hokkaido, Japan; ^2^ Wageningen Greenhouse Horticulture, Wageningen University and Research, Wageningen, Netherlands

**Keywords:** UAV, Potatoes, volume, vegetation indices, multispectral

## Abstract

A phenotyping pipeline utilising DeepLab was developed for precisely estimating the height, volume, coverage and vegetation indices of European and Japanese varieties. Using this pipeline, the effect of varying UAV height on the precise estimation of potato crop growth properties was evaluated. A UAV fitted with a multispectral camera was flown at a height of 15 m and 30 m in an experimental field where various varieties of potatoes were grown. The properties of plant height, volume and NDVI were evaluated and compared with the manually obtained parameters. Strong linear correlations with R^2^ of 0.803 and 0.745 were obtained between the UAV obtained plant heights and manually estimated plant height when the UAV was flown at 15 m and 30 m respectively. Furthermore, high linear correlations with an R^2^ of 0.839 and 0.754 were obtained between the UAV-estimated volume and manually estimated volume when the UAV was flown at 15 m and 30 m respectively. For the vegetation indices, there were no observable differences in the NDVI values obtained from the UAV flown at the two heights. Furthermore, high linear correlations with R^2^ of 0.930 and 0.931 were obtained between UAV-estimated and manually measured NDVI at 15 m and 30 m respectively. It was found that UAV flown at the lower height had a higher ground sampling distance thus increased resolution leading to more precise estimation of both the height and volume of crops. For vegetation indices, flying the UAV at a higher height had no effect on the precision of NDVI estimates.

## 1 Introduction

The precise monitoring of the phenotypic properties of potatoes is important for the development of new potato varieties for high quality and improved yield. Not only are some varieties susceptible to disease infestation such as early blight disease but also, their growth properties affect the yield of potatoes. Until today, monitoring of the growth of crops has relied on manual physical sampling where height of potato crops is sampled in the plots containing the phenotypes. However, this is not only limited to a small area and several crops within the plots, but also the estimation of the height of the crops is difficult, especially due to the complex canopy of potato crops.

Recently the utilisation of remote sensing techniques together with image processing has enabled the precise estimation of the phenotypic properties of potatoes. Especially, the utilisation of UAV’s has revolutionized phenotyping not only due to their low cost, but also a large area can be sampled in a short time. The emergence of potato crops was estimated using UAV thus reducing the time required for manual sampling of potato crops to determine their emergence (Li et al., 2019). The disease severity on potato crop field was estimated using an RGB camera mounted on a UAV (Sugiura et al., 2016) and while Jindo et al. (2023) utilised UAV to detect the damage caused by potato cyst nematode, Van De Vijver et al. (2022) utilised UAV to detect early blight disease. Furthermore, a multispectral camera mounted on a UAV could estimate SPAD values of barley leaves as demonstrated by Liu et al. (2021). The prediction of yield has also been made possible by utilisation of UAV in combination with machine learning (Sun et al., 2020), and also by combining cultivar information (Li et al., 2021).

Hitherto, parameters of plant height and canopy cover estimates have been used to measure the ability of crops to intercept radiation and also as a representation of the activity of the crop that relate to growth and development. Stewart et al. (2007) found a high correlation between canopy cover and leaf area index (LAI). Using non-linear models, Bojacá et al (2011) could estimate potato crop canopy coverage on different fields. However, only a small area could be sampled hence limiting observation in the whole field. de la Casa et al. (2007) found that there was a high linear correlation between potato crop coverage and the fraction of light interception. However, (Boyd et al., 2002) found that such a correlation of crop coverage with LAI, although linear, it varies with management of the field. Furthermore, (Bojacá et al., 2011) found that applying a non-linear model to characterise potato canopy coverage could yield more precise simulation results. Using crop canopy, Li et al. (2019) investigated the rate of emergence of various varieties and assessed the differences in the uniformity of their emergence from the soil. However, the penetration of light is affected not only by the crop surface cover, but also the architecture of the canopy, leaf size, angle, and the number of leaves. Photosynthesis activity is highly related to the light intensity within the leaf canopy, which is depended on the leaf size, distribution, and volume of the canopy. Therefore, it is important to estimate not only the surface crop cover but also the 3D properties of the potato canopies for informed decision making.

To generate such values, Structure from Motion (SfM) is utilised to reconstruct the images to form a 3D model. This is normally used in UAV imagery since special active illumination is not required. Furthermore, the resolution of the constructed model depends not only on the number of images, but also on the ground sampling distance and the resolution of the acquired images (Paulus, 2019). While, the combination of crop height and coverage was used to determine the spraying volume on potato (Xie et al., 2022), it was found that the point cloud data was unreliable especially during low coverage. Burgess et al. (2017) utilised individual plants extracted from a field grown plot to generate 3D representation of crops. However, this is difficult to apply in phenotyping in the actual field. Furthermore, due to the complex canopy of the potato crops and the ridges planted on them, it is imperative to develop an improved technique for extracting phenotypic properties of potato crops.

Hitherto, an easy-to-use automatic system for generating important crop properties like height, volume, crop coverage and vegetation indices has not been developed. This has limited such analysis to either commercial software or complicated programming skills which not only require fundamental understanding, but also complex computer environmental settings. Guo et al. (2017) developed a python-based tool for phenotyping. However, this could only estimate ground coverage ratio. Tresch et al. (2019) developed an automatic technique for generation of plots in the field thus making it easier to divide the field into plots. It is imperative to estimate not only the coverage but also the height and the volume of potato crops during growth. Especially during emergence, it is important to determine these parameters to precisely determine the sprout rate and their traits. It has been shown that increased flight altitude results in decreased resolution of the densified surface model (DSM) which is used to extract height and related parameters (Abou Chakra et al., 2020). While increasing the UAV flight height reduces the time taken to take mages in the same field, the lesser number of images captured reduces the processing time. This results in reduced time taken during the SfM (Structure from Motion) reprocessing to produce densified surface model and orthomosaic which are used to extract the parameters of height and coverage respectively. However, the increased UAV flight height results in decreased resolution, and this might affect the accuracy of extraction of the parameters of plant height, volume, and coverage. The effect of varying UAV height on seedling rapeseed found that higher GSD resulted in lower precision of NDRE vegetation index when estimating LAI (Zhang et al., 2020). While the effect of varied resolution by resampling the orthomosaic found that high resolution had higher correlation with potato’s above ground biomass, however, this was only limited to a fixed UAV height of 20 m height (Liu et al., 2022). Therefore, it is paramount to determine the best UAV flight conditions that reduce processing time without compromising on the quality of the crop parameters to be estimated. Furthermore, for precise phenotyping properties, it is required to estimate the height, volume and crop coverage of potato varieties with high precision.

In this study, we will develop a new system for estimating the potato varieties traits. Firstly, a new automatic system for processing UAV-obtained DSM and orthomosaic will be developed. Secondly, using this new system, the crop properties of height, volume and coverage of both European and Japanese varieties will be estimated. As a lead on determining the ideal flight parameters for precisely obtain these phenotypic traits, we will compare the accuracy of UAV-obtained data by comparing the precision effect when UAV is flown at two different heights over the same area while analysing the growth parameters of plant height, volume, crop coverage and vegetation indices.

## 2 Materials and methodology

### 2.1 Experimental field set up

**Figure 1:**
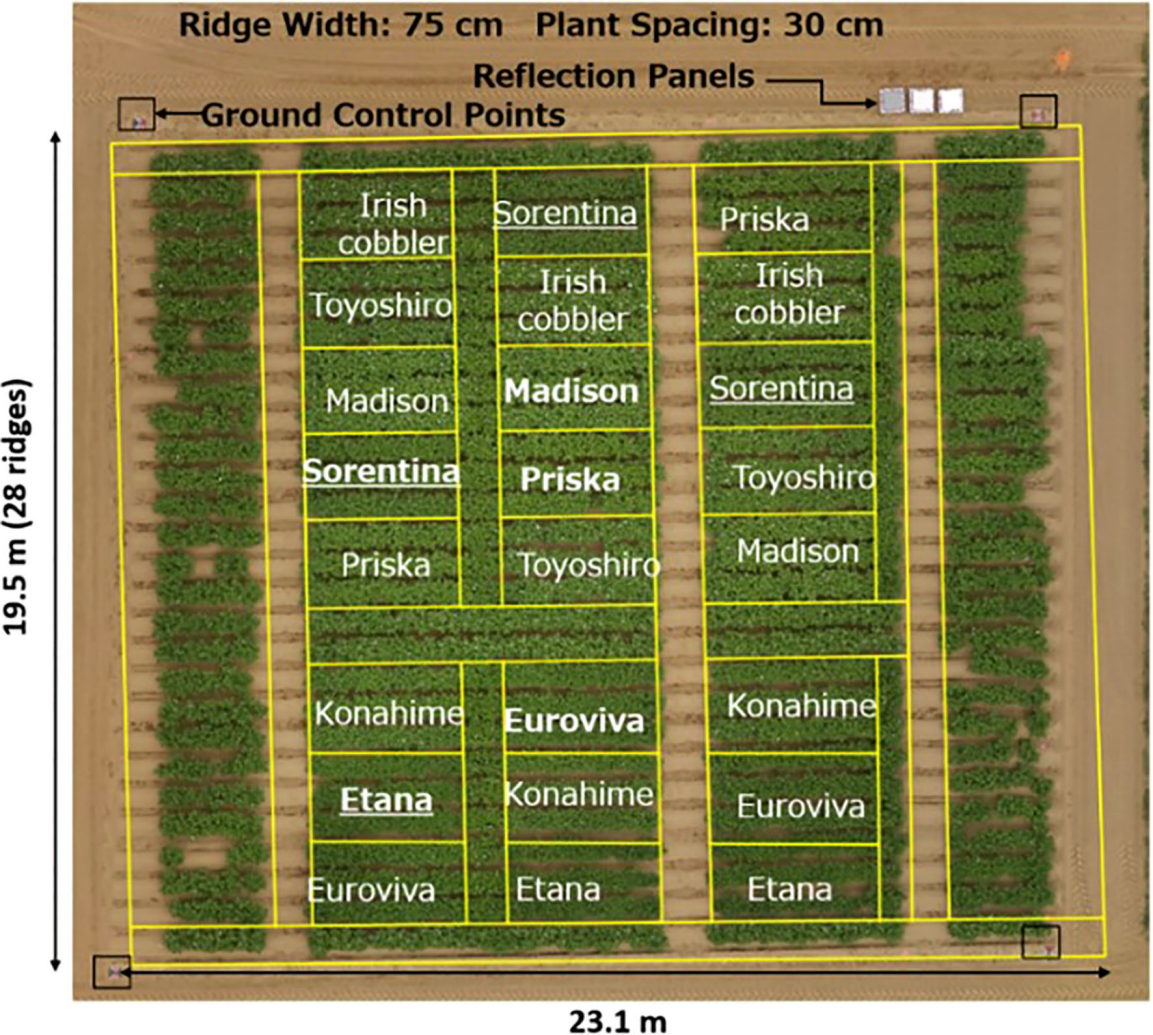
Set up of the field with the varieties planted on plots with three replications and ground control points were placed as shown with a black and red two-tone marker.

Eight varieties of potatoes were planted in an experimental plot in Memuro Hokkaido, Japan inside the National Agricultural and Research Centre (NARO) experimental field station as shown in **Figure 1** on the 7^th^ of May 2021. The experimental field measured 23.1 m by 19.5 m and three replications were planted with each plot measuring 4.5 m by 2.25 m. The potato varieties consisted of 5 European varieties, Euroviva, Etana, Priska, Sorentina and Montana, and 3 Japanese varieties, Toyoshiro, Konahime and Irish cobbler (locally known as Danshaku-imo). The potatoes which were hand planted were firstly cut into two and the half-cut potatoes whose sprout faced upward were placed at a crop spacing of 30 cm and each row was spaced at 75 cm from each other. After planting, the rows were covered with soil using a tractor-driven hiller thus ensuring a ridge of 30 cm height and a spacing of 75 cm between the ridges.

### 2.2 UAV images acquisition

**Figure 2:**
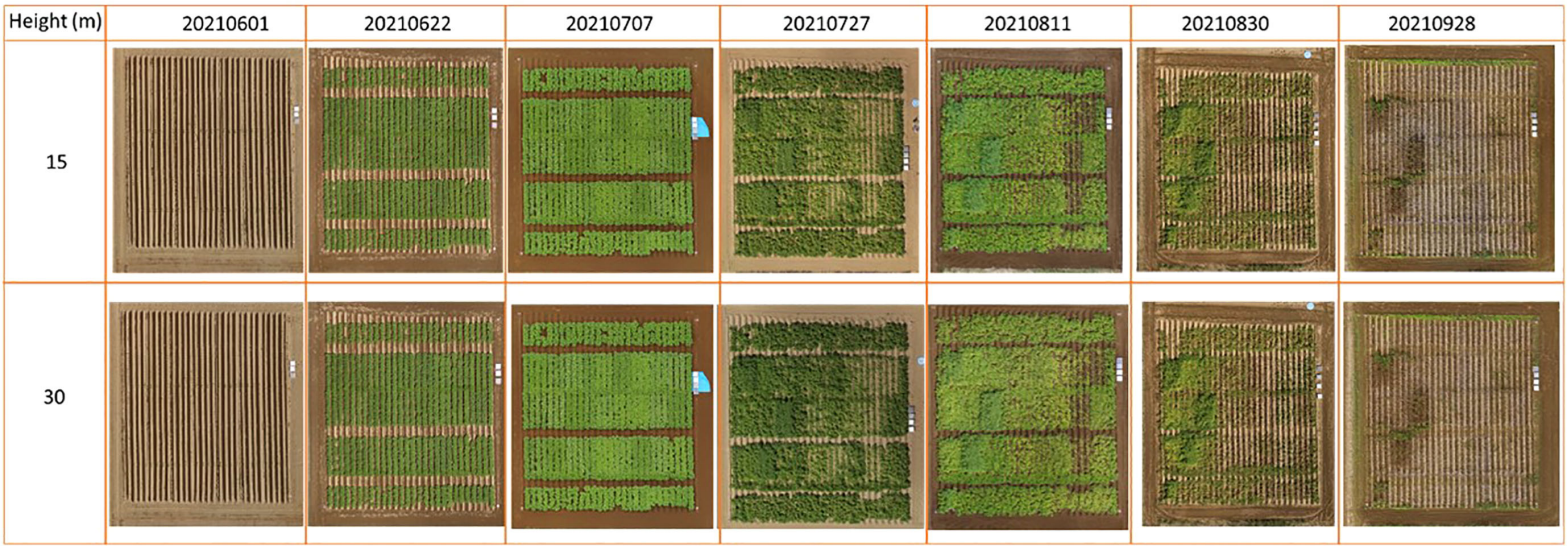
Time series orthomosaic images of the potato varieties plots for both UAV heights of 15 m and 30 m.

For obtaining images on the experimental field from both 15 m and 30 m heights, a DJI P4 Multispectral UAV (SZ DJI Technology Co., Ltd) was utilised. The UAV has a camera consisting of 1 RGB camera and a 5-band multispectral camera. The multispectral camera consists of the Blue (450 ± 16nm), Green (560 ± 16nm), Red (650 ± 16nm), Red-edge (730 ± 16nm) and Near-infrared bands (840 ± 26nm). The UAV which also has an integrated RTK module was set at a FIXED GNSS position thus ensuring precise positioning when taking images. Furthermore, four ground control points were set at the four corners of the experimental field and their coordinates measured thus ensuring the project was georeferenced. The flight plan consisted of taking images in a grid system both at 15 m and 30 m. The time series images when the UAV is at 15 m and 30 m is as shown in **Figure 2**. For both heights, the RGB camera and the multispectral cameras ISO was set to AUTO mode with the shutter speed set at 1/1000 and shooting interval of 2 seconds with a front and side overlap of 80%. The camera was set such that the shooting angle was perpendicular to the course, and the cameras were set at Nadir (facing downwards) at an angle of 90 degrees with the horizontal field plane.

### 2.3 Pre-processing of the UAV images

The images were aligned and processed using a SfM (Structure from Motion) technique where the DSM was generated from the two-dimensional images obtained from the UAV. To do this, Pix4D mapper (Pix4D SA) software was utilised. In order to increase the accuracy of the map, the pre-measured GCP (Ground Control Points) were imported and marked in the ray cloud. Finally, re-optimisation was carried out, resulting in a calibration error of ± 2 cm. This error is equal to the absolute error of the RTK measurement of the GCP’s. The point cloud was processed after which the orthomosaic and the Digital Surface Model (DSM), the Orthomosaic (RGB) and the reflectance maps (for each sensors’ wavelength) were generated.

The parameters for obtaining UAV images and processing time when processing using a GPU NVIDIA Quadro RTX 6000 with an intel core i9 and a CPU processing speed of 3.31GHz are compared as shown in **Table 1** below.

**Table 1:** Comparison of flight parameters for both flight altitudes.

Height (m)	Camera angle	Mission type	Flight Time (min)	Processing time DSM and Orthomosaic
15	Nadir	Parallel	15	3 hours
30	Nadir	Parallel	7	1 hour

### 2.4 Plant segmentation

**Figure 3:**
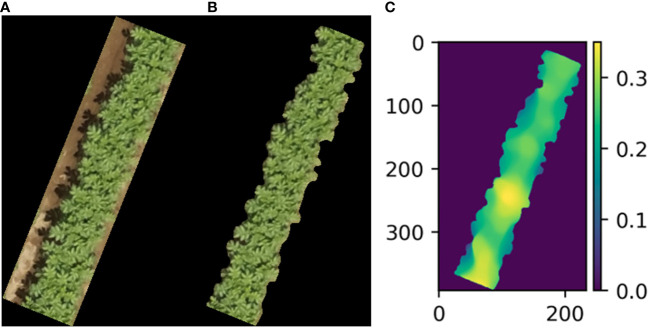
Example image of input image **(A)**, segmented image **(B)** and visualized height map, with height in meters **(C)** obtained on 46^th^ day after planting.

To do determine the crop properties, the crop parameters must be segmented from the bare soil. In this research segmentation is done by using DeeplabV3+ implemented in detectron2 (Chen et al., 2018). DeepLab is a deep learning based semantic segmentation method (Wu et al., 2023). In this research we used the pretrained weights available from the detectron2 framework. Transfer learning was applied by annotating 48 potato images. Since the dataset was quite small the number of iterations was set to 1000. Image size was set to 384 x 384. All the other parameters were similar as those mentioned by Wu et al. (2023). An example of the segmentation on an independent test image is shown **Figure 3**. Other segmentation methods like Otsu thresholding (Li et al., 2019) would fail at sunny condition or when the image is fully covered by crops.

### 2.5 Plant height measurement

**Figure 4:**
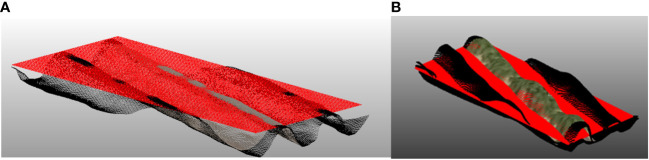
**(A)** plot with the fitted plane (red) on bare soil (day 5 after planting) and **(B)** 36 days after planting.

A densified surface model with a resolution of 0.833 cm/pixel and 1.667 cm/pixel was generated from the densified point cloud for images taken at 15 m and 30 m respectively. To determine the height of crops from each plot, shapefiles measuring 3 m by 0.75 m were generated as representatives of each variety’s plot’s area (using QGIS software). To ensure precise height estimation, a plane surface on the top of the ridge was generated for each plot. This was done by determining the height of the bare soil 10 days after planting. Noise was removed by sorting the height array and selecting the height at 90% of the data. This base altitude ( $Zplane_{i}$ ) was determined for each plot as shown in **Figure 4**.

In the successive days as the crops grew, the height of the crops increased. The height was calculated by segmenting the background from the image and subsequently the difference between the base altitude and the terrain altitude of each segmented plot (Z_y_) was used to determine the height, as shown in Eq. 1. Where i indicates the plot number and j indicates the pixel number.


(1) $$H_{ij}=Z_{ij}-Zplane_{i}$$

The height of the crops in each plot was extracted by determining the average of the total number of pixels.

As a proof of concept, the height of crops from each crop was measured manually on a weekly basis. This was done by placing the meter rule at the top of the ridge and measuring the height of the crop close to the apex. Taking care to prevent injuring the potato crops foliage, the height of the 5 sample crops in each plot were measured recorded from which the average height with growth period was calculated.

### 2.6 Volume measurement

The volume of the crops from each plot was determined by summation of the total volume of the pixels from the densified surface model. Each pixel consisted of length *L_ij_* (cm), the width $W_{ij}$ (cm), and the height $H_{ij}$ (cm). The height was obtained using Eq. 1 (section 2.4). By multiplying the height with the length and width of each cell the volume was calculated as shown in Eq. 2 below.


(2) $$V_{ij}=L_{ij}\;\times W_{ij}\times H_{ij}$$

The length and width of each pixel is equidistant and is equal to the ground sampling distance (GSD). Therefore, the length and width of each pixel obtained from the UAV’s height, i.e., 15 m and 30 m was 0.833 cm/pixel and 1.667 cm/pixel respectively. Although the grid of the higher GSD i.e., 30 m would be larger than the grid with a smaller GSD, however, since the total volume was limited to the plot area of each variety, then the total area utilised for estimating volume would be similar despite their different GSD.

In order to compare the accuracy of estimation, the volume of the crops was also estimated by hand. This was done by manually measuring the width, breadth, and height of four samples from each plot. The average volume per plot was estimated from the four samples and used as the ground truth values for the volume of the crops.

### 2.7 Vegetation indices measurement

To determine the health of the potato crops during growth, each plot’s average Normalised Difference Vegetation Index (NDVI) was estimated (Datt, 1999) from the NIR and Red reflectance maps generated from the processing of images in these respective wavelengths. In the mid to late stages when the chlorophyll concentration was relatively high, the Normalised Difference RedEdge (NDRE) was utilised to estimate the health of the potato crops. Both the NDVI and NDRE are popularly used to map the variability of nitrogen and hence determine the fertilizer requirement. The ratio of the reflectivity in the NIR and red-edge bands were used to estimate the chlorophyll content in the leaves using the Chlorophyll index -Red-Edge (Clre). Leaf Chlorophyll Index (LCI) was used to determine the change in chlorophyll content by estimating the spectral reflectance properties of both the near-infrared and the red reflectance wavelengths. The indices are shown in **Table 2**.

**Table 2:** Vegetation indices generated from the respective reflectance maps.

Vegetation index	Equation	Reference
Normalised Difference Vegetation Index (NDVI)	$\frac{NIR-Green}{NIR+Green}$	(Datt, 1999)
Normalised Difference Red Edge (NDRE)	NIR−RedEdgeNIR+RedEdge	(Barnes et al., 2000)
Chlorophyl Red Edge (Clre)	NIRRedEdge−1	(Gitelson, 2005)
Leaf Chlorophyll Index (LCI)	NIR−RedEdgeNIR+Red	(Rouse et al., 1974)

To compare the estimated NDVI values obtained from the reflectance maps generated by the UAV images, a handheld crop sensor, GreenSeeker (Trimble Inc.) was utilised to measure NDVI. The sensor had sensitivity of measurement at the wavelengths of Red 660 ± 10 nm (full-width half-magnitude) and near-infrared 780 ± 15 nm (full-width half-magnitude). The sensor emits both of these wavelengths and measures the amount of each wavelength reflected from the surface of the crops. Measurements were taken by engaging the trigger and scanning the potato field crops’ plots at height of about 50 cm from the surface of the crops. In each plot, the reading was taken three times and the average value was calculated.

## 3 Results

### 3.1 Potato crop height estimation

**Figure 5:**
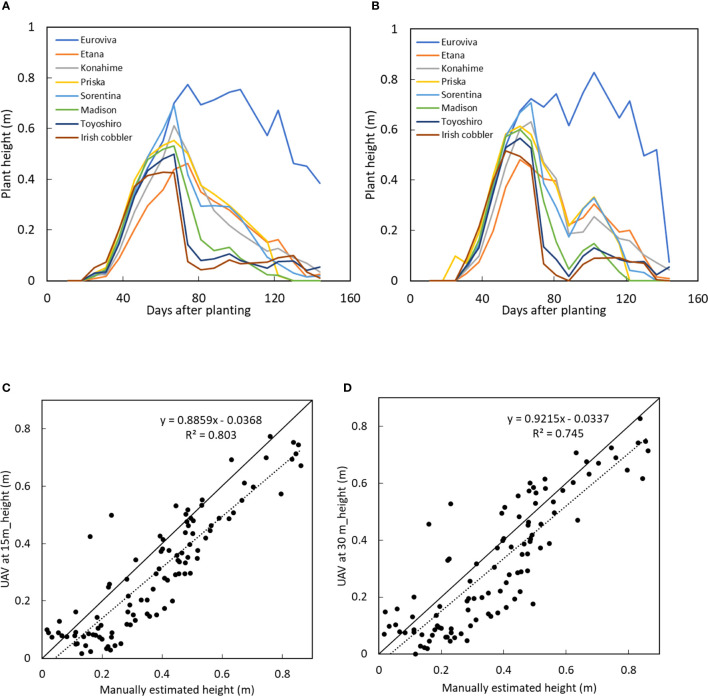
Plant height of 8 varieties with growth time when the UAV was at **(B)** 15 m and **(C)** at 30 m and comparison between manually measured plant height with the UAV-obtained height when the UAV was at **(C)** 15 m and **(D)** at 30 m.

The orthomosaic images and the height of the potato crops was obtained after processing the densified surface model obtained when the UAV was flown at 15 m and 30 m as shown in **Figures 5A, B** below. During the early growth stages, there was a gradual increase in the height of the crops especially after emergence. However, 30 days after emergence, there was a sporadic increase in the height of all the potato varieties. The growth peaked between 60-67 days after planting from which the plant height decreased as the potato crops matured and senescence started. However, for the Euroviva variety, the plant height continued to increase with a prolonged growth period with the growth peaking at 102 days after planting after which maturity and senescence occurred.

For both UAV-heights, the plant height peaked between day 61 day 67. However, during the senescence stage, it was observed that plant heights obtained when the UAV was at 30 m showed a gradual increase in plant height at day 102 while the plant height obtained when the UAV was at 15 m showed a slight or no increase in plant height during the senescence stage. At days 81 and 102 where a large decrease followed by a rapid increase in crop was observed, the standard deviation showed that a larger error was observed in the height values obtained when the UAV was at 30 compared to at 15 m as shown in **Tables 3**, **4**.

**Table 3:** STDEV comparing plant heights on day 88.

Variety name	15 m	30 m
Euroviva	0.005	0.033
Etana	0.073	0.079
Konahime	0.069	0.097
Priska	0.010	0.035
Sorentina	0.036	0.045
Madison	0.033	0.030
Toyoshiro	0.029	0.032
Irish cobbler	0.018	0.00

**Table 4:** STDEV comparing plant heights on day 102.

Variety name	15 m	30 m
Euroviva	0.005	0.055
Etana	0.038	0.051
Konahime	0.021	0.063
Priska	0.019	0.054
Sorentina	0.020	0.060
Madison	0.025	0.048
Toyoshiro	0.029	0.043
Irish cobbler	0.012	0.018

**Figure 6:**
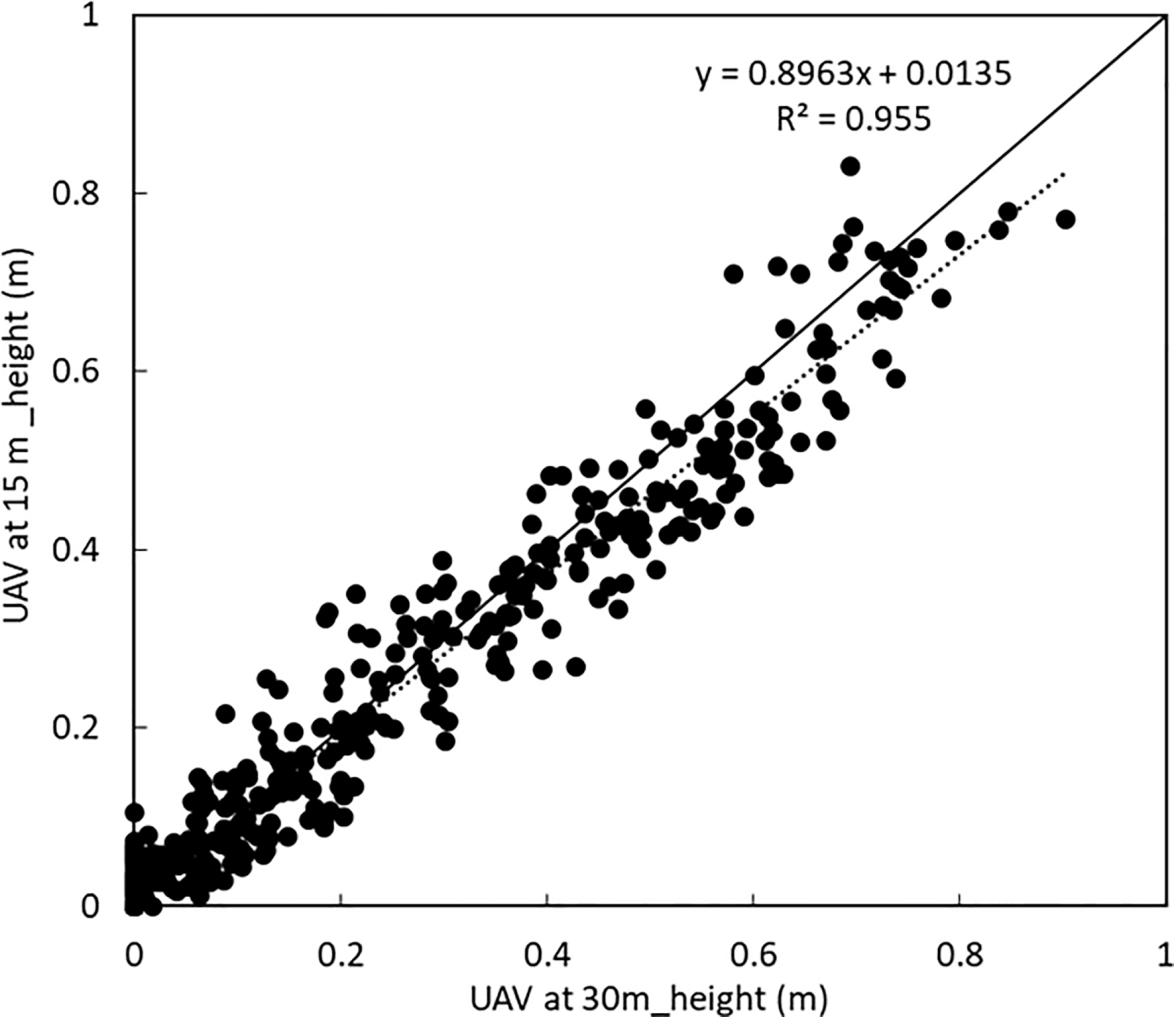
Comparison between plant height of all varieties when the UAV was at 15 m and at 30 m. A linear correlation with a coefficient of variation of 0.947 was obtained.

There was a high correlation between the plant height obtained when the UAV was at 15 m and at 30 m as shown in **Figure 6** below.

It was clear that especially during the early growth (plant height 0 - 0.1 m), the plant height obtained from images taken at 30 m showed no change in the height of the crops while there was observable change and differences in the height of potato crops when the UAV was at 15 m as shown in **Figure 5C**. The differences in the plant height were also compared with the manually obtained plant height obtained by sampling in the plots of the respective varieties. It was observed that while the plant height obtained from the UAV at 15 m showed a high linear correlation with the manually obtained values, the plant height obtained when the UAV was flown at 30 m as shown in **Figure 5D**. had a slightly lower correlation with the manually obtained plant height.

### 3.2 Plant volume estimation

**Figure 7:**
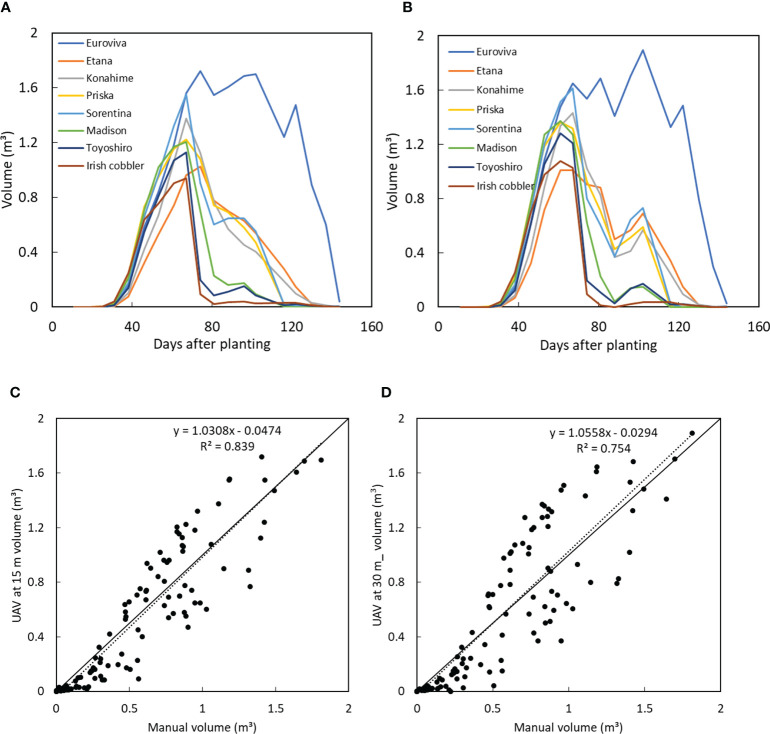
Plant volume of 8 varieties when the UAV was at **(A)** 15 m and **(B)** at 30 m and comparison between manually measured plant volume with the UAV-obtained volume when the UAV was at **(C)** 15 m and **(D)** at 30 m.

The potato varieties’ volume was compared between those estimated when the UAV was at 15 m from those obtained when the UAV was at 30 m as shown in **Figures 7A, B**. A similar tendency of growth was obtained when the UAV was at both heights. During the early stages of sprout emergence, there was little or no observable change in the volume of the potato varieties, until day 30, after which a rapid increase in the volume of the potato varieties was observed. From the volume estimated when the UAV was at 15 m, it was observed that the volume of all the varieties peaked at day 67 while the volume estimated when the UAV was at 30 m peaked at day 61. A similar growth curve tendency was obtained in both data sets when the UAV was flown at 15 m and at 30 m.

**Figure 8:**
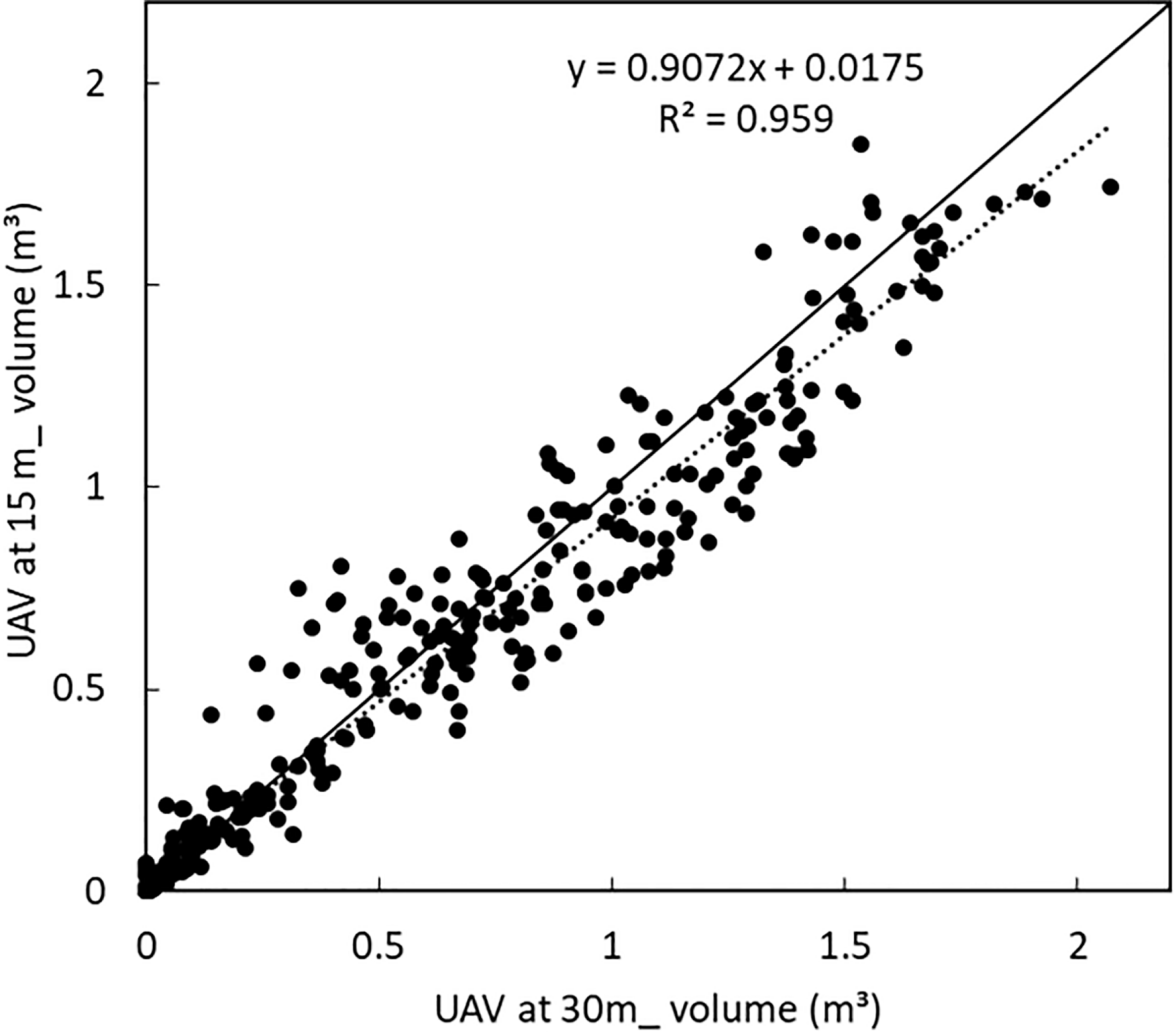
Comparison between plant volume of all varieties when the UAV was at 15 m and at 30 m. A linear correlation with a coefficient of variation of 0.956 was obtained.

A high linear correlation was obtained between the volume of all the varieties when the UAV was at 15 m and at 30 m as shown in **Figure 8**. While it takes less time to obtain images at a higher altitude (30 m in this case), resulting in higher ground sampling distance and thus lower sampling resolution, it would, however, still have sufficient accuracy for precise estimation of the volume of potatoes with similar precision as that obtained when the UAV is flown at half the height (15 m).

A comparison was made between the UAV estimated volume and the manually estimated volume. From the manual measurement, the height, width and length of the potato crops was measured as a representative of the volume of crops. While a high linear correlation was obtained between the UAV estimated volume when the UAV was at 15 m, a slightly lower correlation was obtained from the volume estimated when the UAV was at 30 m as shown in **Figures 7C, D** respectively. However, in both cases, the manually estimated volume was higher than the UAV estimated volume.

**Figure 9:**
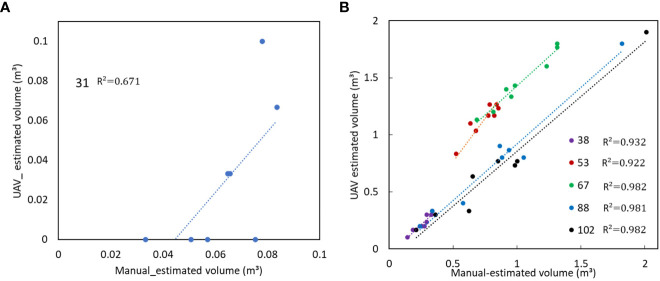
Early stages of growth **(A)** 31^st^ day after planting and successive days **(B)** 38 to 102 days after planting showing the comparison between UAV estimated and manually estimated volume.

It was considered that during the early stages of growth, at days 31, there was a low correlation with an R^2^ of 0.67 between the UAV-estimated volume and the manually estimated volume as shown in **Figure 9A**. With successive growth, a high linear correlation of 0.9 was obtained between the UAV-estimated and the manually estimated volume as shown in **Figure 9B**.

### 3.3 Vegetation indices

**Figure 10:**
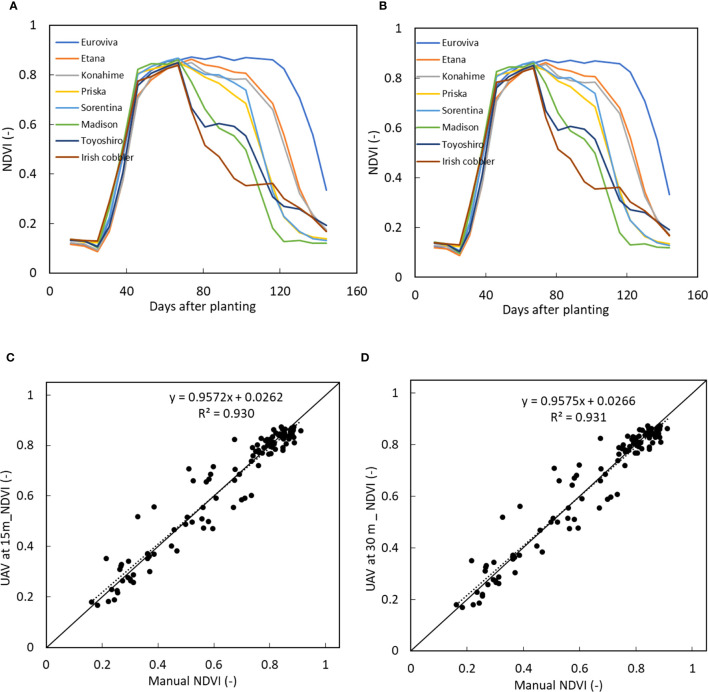
Time series NDVI values of 8 varieties when the UAV was at **(A)** 15 m and **(B)** at 30 m and comparison between manually measured NDVI with the UAV estimated NDVI when the UAV was at **(C)** 15 m and **(D)** at 30 m.

From the multispectral bands, the reflection maps of NIR and Red bands were utilised to estimate the NDVI values of all the varieties of potato crops and a comparison was made between the NDVI values obtained when the UAV was at 15 m and 30 m as shown in **Figures 10A, B** respectively. A similar tendency of change in NDVI was obtained between the two datasets where the NDVI value increased rapidly from 30 days after planting to about 74 days after planting after which the values decreased, except for Euroviva variety which had a prolonged stable NDVI value from day 74 to day 116 after which NDVI values decreased.

**Figure 11:**
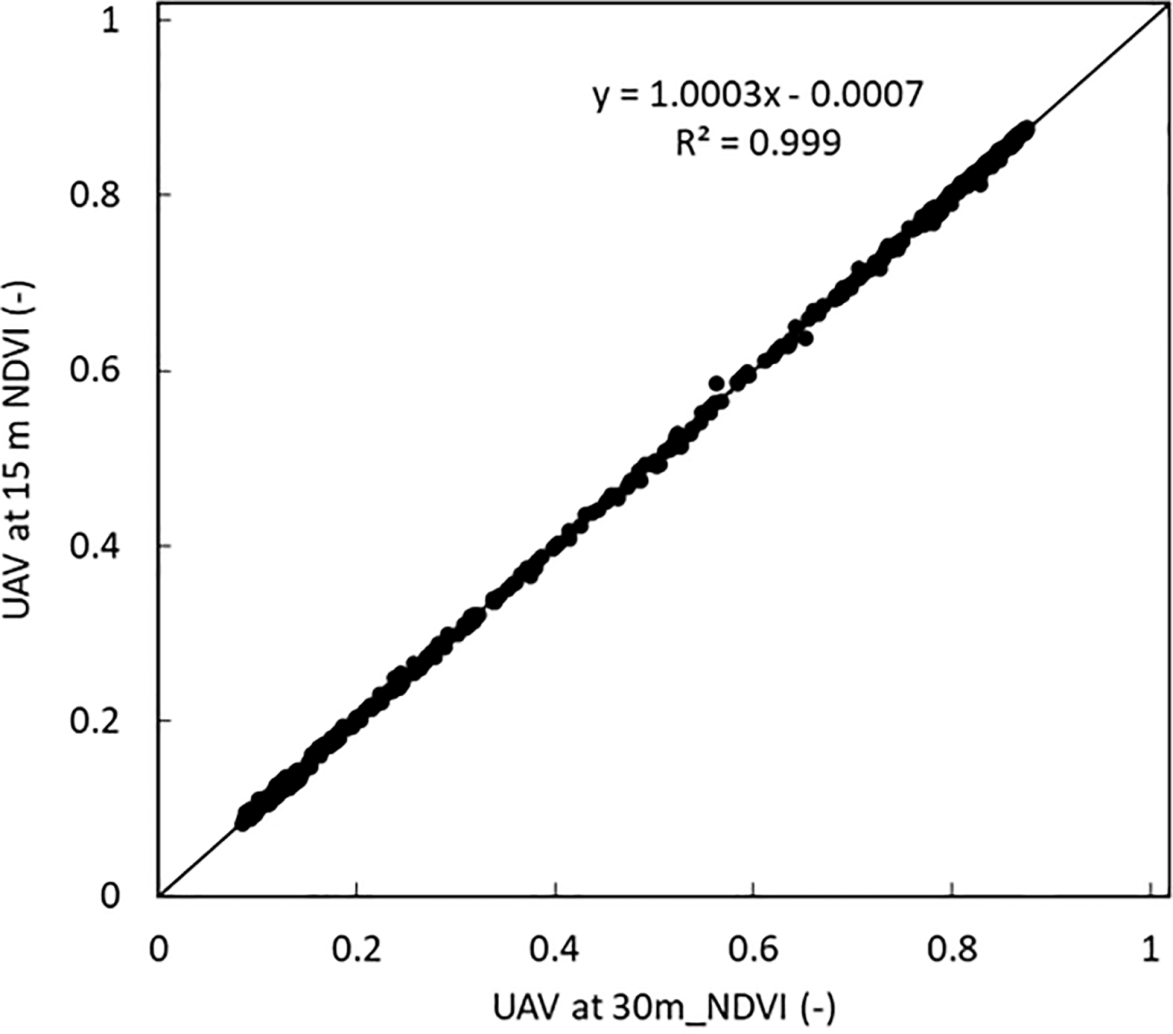
Comparison between NDVI volume of all the potato varieties when the UAV was at 15 m and at 30 m. A linear correlation with a coefficient of variation of 1 was obtained.

A high linear correlation with a coefficient of variation close to 1 was obtained between the NDVI values obtained when the UAV was at 15 m and at 30 m as shown in **Figure 11**.

A comparison was made between the UAV estimated NDVI and the manually estimated NDVI. A similar high linear correlation with a coefficient of variation of 0.97 was obtained between the manually estimated and the UAV estimated NDVI when the UAV was at 15 m and at 30 m as shown in **Figures 10C, D**.

A high coefficient of determination was obtained for all parameters of height, volume and NDVI when the UAV was flown at both heights as shown in **Table 5**.

**Table 5:** Correlation between UAV parameters between UV flown at 15 m and 30 m.

Parameters	R^2^	Adjusted R^2^	RSME	*p* value
Height	0.955	0.955	0.046	0
Volume	0.959	0.959	0.100	0
Coverage	0.992	0.992	0.037	0
NDVI	0.999	0.999	0.004	0

While both UAV-heights can be used to obtain the parameters of height, volume and NDVI, a comparison between manually estimated values of height, volume and NDVI with those when the UAV was at 15 m and 30m was obtained as shown in **Tables 6**, **7** showed that higher precision was obtained when the UAV was flown at a lower height. A high linear coefficient of determination with a lower RMSE was obtained when the UAV was at a lower height of 15 m than when flown at 30 m.

**Table 6:** Comparison between manually estimated and UAV-estimated values at 15 m height.

Parameters	R^2^	Adjusted R²	RMSE	*p*value
Height	0.803	0.801	0.0919	<0.001
Volume	0.828	0.825	0.209	<0.001
NDVI	0.939	0.929	0.060	<0.001

**Table 7:** Comparison between manually estimated and UAV-estimated values at 30 m height.

Parameters	R^2^	Adjusted R^2^	RMSE	*p*value
Height	0.745	0.742	0.113	<0.001
Volume	0.735	0.732	0.280	<0.001
NDVI	0.931	0.930	0.060	<0.001

## 4 Discussion

**Figure 12:**
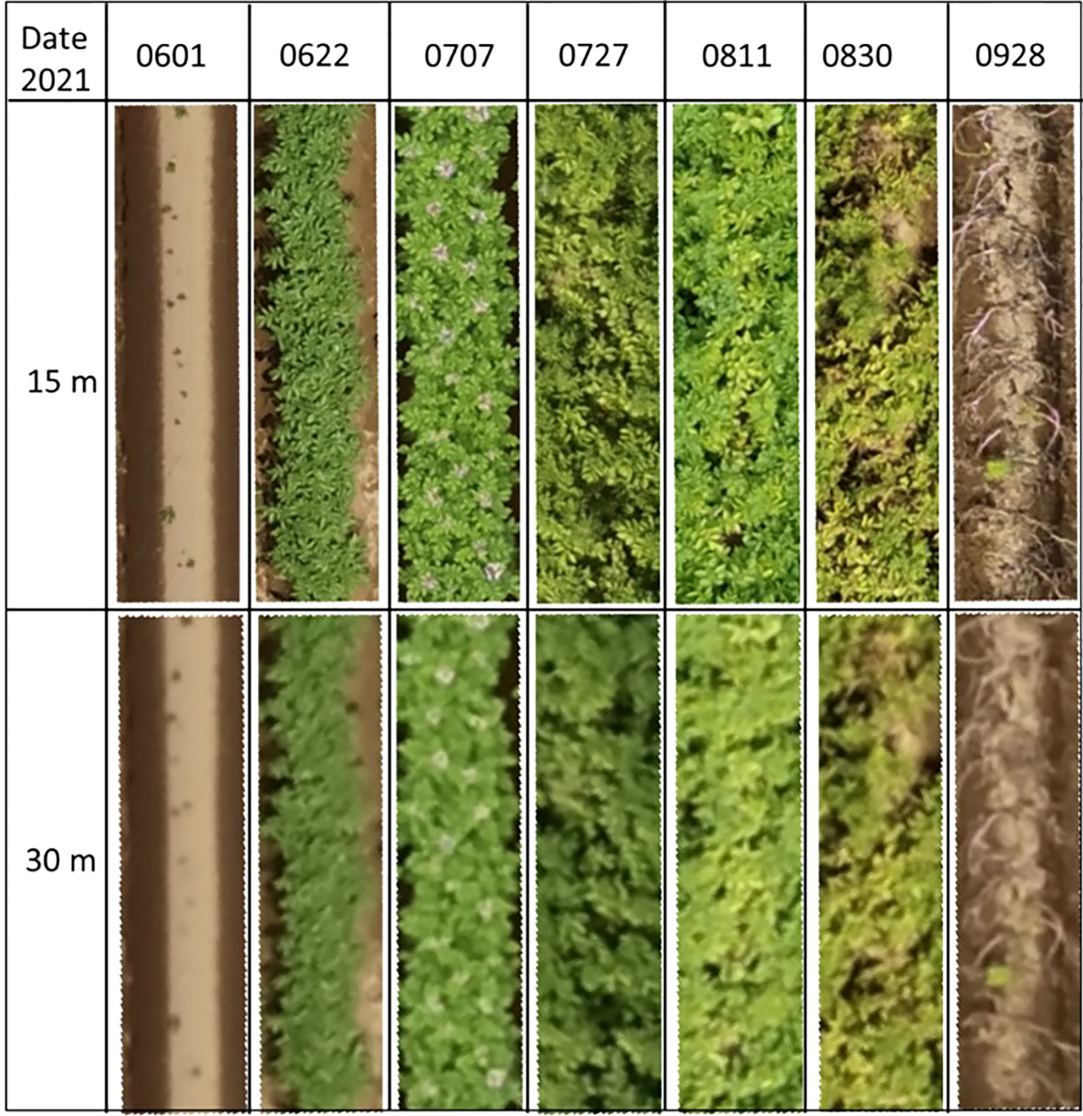
Time series orthomosaic images of one of the plots showing the difference between 15 m and 30 UAV processed orthomosaic files.

Although there was a high correlation between the height of the varieties at both UAV heights, the potato crops height obtained when the UAV was flown at a higher height showed slightly higher errors especially at day 88 and day 102. This was as a result of noise in the densified point cloud as a result of reduced spatial resolution leading to inaccuracies in plant height extraction during the early stages (Moeckel et al., 2018). When the images were obtained at a lower height of 15 m this increased resolution, thus indicating that the plant height increased gradually according to the growth period as shown in **Figure 12**. There was no effect of downwash form the propellers of the UAV at both 15 m and 30 m height. While flying lower than 15 m would increase the ground sampling distance, however, this would also lead to more images and thus lengthening the processing time. Furthermore, for larger fields, this would mean that due to lower ground sampling distance, images would have to be taken at a hover-and-capture mode which takes much longer time as the UAV would have to stop while taking images before proceeding. While flying at higher heights such as 30 m would lead to fewer images and shorter processing time, however increased ground sampling distance leads to lower resolution, thus not ideal especially when estimating the crop traits at the initial stages of growth. Further studies exploring larger phenotypic fields would utilise higher UAV heights in order to cover such large areas.

From the images obtained when the UAV was both at 15 m and at 30 m, Euroviva had the highest height due to its prolonged maturity period. Thus Euroviva plant height remained relatively high even after flowering and the beginning of senescence period. Euroviva which has a late maturity, it continues to grow even after other varieties start to senescence. This was equally translated to the yield as Euroviva has been reported to have higher yields (Seed potatoes of the highest quality, 2021). Due to the prolonged growth period, the light interception area also increased as the height and volume of Euroviva varieties increased. This resulted in increased height and volume of the canopy thus leading to increased light penetration and providing higher sinks for photosynthesis leading to increased photosynthetic activities at the leaves level (Burgess et al., 2017). The sudden decrease in the plant height from day 67 to day 81 for the other varieties during the growth period was a result of senescence where the leaves begin to wither thus leading stems of the potatoes weakening thus decreased height. There was a sudden slight increase in the crops height from day 81 to day 96 as a result of sudden increased precipitation thus leading to the stems vigour increasing and thus increased height, but it was short-lived as withering continued due to senescence and decreased height was obtained until near harvesting period.

Similarly, there was a high correlation between the volume of the potato varieties when the UAV was flown at both heights. It was observed that even during the early stages, since volume estimated not only the vertical elongation but also the horizontal elongation of the canopy, a normal growth curve was observed at both heights. The manual estimated volume was higher than the UAV estimated volume. This is because the manual measurement assumes that the vegetation block is cuboid thus estimates volume using the measurements of height, length and volume, unlike potato canopy, which is not only irregular in shape, but also has air spaces in between the vegetation foliage, thus resulting in overestimation when measured manually. Secondly, in the first days, the potatoes are relatively small. Deep learning networks like DeepLabv3+ are capable of detecting objects, but small objects ten to disappear in the large networks. Small plants are consequently not always recognized causing an underestimation of the volume compared to the manual measurements (**Figure 10A**). In the successive days, a higher correlation was obtained between the UAV and the manually obtained data (**Figure 10B**).

Furthermore, volume would be ideal in monitoring the growth of mixed varieties in large breeding fields since a similar precision for volume estimation would be obtained when the UAV was flown at a higher height thus ideal for obtaining phenotypic properties. Sun et al. (2018) observed that large differences in the volume of the cultivars could be obtained, in comparison to canopy area. Furthermore, while it is erroneous to measure the volume of crops manually due to their irregularity in both shape and structure, volume estimation using the densified surface model was precise and added a new trait for varietal differentiation. A similar tendency in the growth curve NDVI was obtained when the UAV was flown at both heights. This is because the reflectance map was not easily affected by the difference in the UAV height since clear reflectance values were obtained. This shows that the NDVI values are not affected by the changes in the ground sampling distance hence a higher UAV height would be ideal as it saves time and battery when taking images in the field. Furthermore, a similar high precision would be obtained thus enabling faster return and monitoring of potato varieties in the field.

However, because of the difference in the NIR bands between the UAV -multispectral camera (840 ± 26 nm) and the GreenSeeker (780 ± 15 nm) the sensitivity of measurement of NDVI varied between the former from that of the latter. Since the UAV utilises the sunshine sensor to correct for the irradiation, it is ideal in extracting the reflectance values on different days where the radiation would vary. Furthermore, since sampling using the GreenSeeker can only be done in a single plot, then utilisation of the UAV would be ideal in determining the change in the vegetation indices over the whole plot. Therefore, not only would it be faster to monitor a larger field using UAV compared to manual sampling of NDVI which can only be done in a small area and take a lot of time for sampling measurement, NDVI can be estimated using UAV at a faster rate, lesser time and the whole field can be estimated thus ensuring precise monitoring of the growth of potato varieties in the field. A combination of these parameters of plant height, volume and NDVI would be ideal for predicting yield of potato crops or estimating nitrogen content (Lu et al., 2021).

## 5 Conclusion

A precise phenotyping pipeline using DeepLab was developed for estimating the potato crop traits from both the European and Japanese potato varieties. Using this pipeline, the precise estimates of height, volume and vegetation indices of potato varieties was compared with UAV-images taken at 15 am and 30 m altitude. There was a high correlation between the potato varieties at both heights with an R^2^ of 0.947, 0.956 and 1 for crop varieties height, volume and NDVI respectively. While a high correlation was obtained, it was found that in the early stages of growth, higher resolution obtained from 15 m would be ideal for determining the volume and height of the potato crops after emergence. Furthermore, for NDVI, there was no difference between images obtained at the two heights of the UAV. As a proof of concept, it was found that there was a high correlation between the UAV obtained parameters of height, volume and NDVI with those measured manually with R^2^ of 0.856, 0.845 and 0.968 respectively when the UAV was flown at a height of 15 m thus confirming the preciseness of the parameters obtained by the UAV. In the future, it is expected that these parameters would not only be useful for yield prediction but also provide breeders with more information about the varieties in the field thus saving time compared to manual measurements.

## Data availability statement

The original contributions presented in the study are included in the article/supplementary material. Further inquiries can be directed to the corresponding author.

## Author contributions

SN wrote the first draft of the manuscript. ST contributed to statistical analysis. BM and GP contributed to data analysis. KT and HT contributed to conception and design of the study. All authors contributed to the article and approved the submitted version.
